# Light and focused ion beam microscopy workflow for resin-embedded tissues

**DOI:** 10.3389/fcell.2023.1076736

**Published:** 2023-01-25

**Authors:** Horacio Merchant-Larios, David M. Giraldo-Gomez, Adriana Castro-Dominguez, Alejandro Marmolejo-Valencia

**Affiliations:** ^1^ Instituto de Investigaciones Biomédicas Departamento de Biología Celular y Fisiología, Universidad Nacional Autónoma de México (UNAM), México City, México; ^2^ Carl Zeiss de México S. A. de C. V., Research Microscopy Solutions, , México City, Mexico

**Keywords:** focused ion beam, 3D tissue ultrastructure, resin embedded, large samples, developing mammalian ovaries

## Abstract

Although the automated image acquisition with the focused ion beam scanning electron microscope (FIB-SEM) provides volume reconstructions, volume analysis of large samples remains challenging. Here, we present a workflow that combines a modified sample protocol of the classical transmission electron microscope with FIB-SEM volume imaging. The proposed workflow enables efficient 3D structural surveys of rabbit ovaries collected at consecutive developmental stages. The precise trimming of the region of interest adds the time dimension to the volume, constructing a virtual 4D electron microscopy. We found filopodia-like processes emitted by oocyte cysts allowing contact between oocytes not previously observed.

## Introduction

Classical transmission electron microscopy (TEM) and 3D electron tomography (3DET) generate the highest resolution 3D images of cells and tissues. However, the resolution of the first 3DET reconstructions depended on theoretical models based on the spacing and angular range of projection images in a tilt series and the signal-to-noise ratio between images (Cardone et al., 2005; Heymann et al., 2008). The empirical data of the classical TEM images depend on the heavy-metal-stained cell and extracellular structures, which produce variable electron density affecting the electron scattering. In this context, the physical thickness of the sections becomes critical for the optimal resolution of the tissue ultrastructure. Moreover, the optimal resolution of 3DET was obtained in pre-irradiated areas of thin sections before the tilt series to prevent shrinkage and mass loss (Perkins et al., 2009). On the other hand, 3D reconstruction of ultramicrotome serial thin sections (around 700 nm thickness) at the TEM resolution level requires intensive operator involvement and is time-consuming. (Davis et al., 1986; Harris, 1999). However, volume scanning electron microscopes (SEMs) opened the possibility of visualizing larger volumes of regions of interest in biological tissues (Peddie and Collinson, 2014; Titze and Genoud, 2016; Ronchi et al., 2021).

There are two main approaches: 1) Serial block-face SEM (SBFSEM) developed by combining a custom-designed ultramicrotome inside a low vacuum SEM, volume reconstructions of plastic-embedded tissues, allowed reconstructions over hundreds of µm with a cell organelle resolution (Denk and Horstmann, 2004). 2) Focused ion beam scanning electron microscope (FIB–SEM) (Heymann et al., 2006; Knott et al., 2008; Hekking et al., 2009) generated the opportunity to acquire images of volumes at nanometer resolution in a semi-automated approach. In the FIB-SEM instrument, an ion beam (often Gallium ions) is used to mill thin layers of material and combine iterative slicing and SEM imaging. The electron beam is scanned onto the recently exposed sample surface to produce high-resolution images of the sample. The iteration of these two steps thousand times thus results in the generation of a series of surface maps of the specimen at regularly spaced intervals, which can be converted into a three-dimensional stack reconstruction of the sample (Narayan and Subramaniam, 2015). The achievable XY resolution of SBFSEM and FIB-SEM is comparable (1–2 nm) (Wei et al., 2012; Müller et al., 2021; Ronchi et al., 2021). FIB-SEM is suitable for automatically acquiring volumes ranging from subcellular to a few cells at high-isotropic resolution.

Even though the SBF-SEB and FIB-SEM identify regions of interest (ROI) in volumes of several hundreds of microns of cut block faces, 3D reconstruction of cell-cell and cell-matrix interactions in tissues of developing embryos requires volumes of mm. Morphological cues can be used alternating ultramicrotome serial semithin sections (1.0 μm) with FIB-SEM. It is unlikely that SEM surface imaging will ever reach the lateral resolution of imaging thin sections of ROI in the TEM located on semithin sections at the light microscope.

The subject of the current article is to illustrate the utility of adapting the classical TEM of sample preparation and handling to volume scanning electron microscopy by combining the large field of view of the light microscope (LM), the higher resolution of TEM, and the volume reconstruction of FIB-SEM. The combined techniques provide a powerful multimode method to study the structural dynamics of tissues in developing organs. The proposed workflow enables efficient 3D structural surveys of organs collected at consecutive developmental stages. The precise trimming of the ROI adds the time dimension constructing a virtual 4D electron microscopy. Here we use the example of rabbit ovaries at different developmental stages.

## Sample preparation combining light microscope with focused ion beam scanning electron microscope volume imaging

To study de changing structure of cells and tissues of developing organs, the actual workflow works well for developing rabbit ovaries. However, the optimal preservation of other organs may require modifying some of the proposed steps. Optimal fixation, heavy metals staining, and hard resin embedding mixture is necessary for large samples’ volume analysis.

Prefixation. To ensure that the fixative reaches the tissue as fast as possible. The best fixation of tissues *in situ* was obtained by perfusing the animals with saline (0.9% NaCl) for 5 min, followed by the Karnovsky solution (2.5% glutaraldehyde, 1% paraformaldehyde in 0.1 M cacodylate buffer. After that, the dissected tissues were immersed in the fixative of Karnovsky containing 0.3% malachite green and left overnight at 4°C. The malachite green acts as a chemical mordant that increases the number of heavy metals in the bloc during sample processing. Osmium tetroxide, lead citrate, and uranyl acetate provide the heavy atoms required to reveal the ultrastructural patterns of organelles, cells, and tissues at TEM and FIB-SEM.

Post fixation. All the solutions were freshly prepared and maintained at 4°C. After the aldehyde fixation, the samples were washed with sodium cacodylate (0.1 M, pH 7.3) for 15 min each. Then, the samples were immersed 2 h. Into 1% OsO4 in Millie-Q water and washed twice in sodium cacodylate buffer, 15 min each.

Dehydration. We found that a series of acetone at 25%, 50%, 75%, and 95% of 15 min each was the best to prevent swelling or shrinkage of tissues.

Embedding: After fixation and dehydration, embedding is the most critical step for the current sample protocol. We used Epon 812 according to Luft’s (1961) two different mixtures to vary the hardness of the blocks. The original proportions of the mixtures were somewhat modified: Mixture A. Epon 812, 62 mL; Dodecenyl succinic anhydride (DDSA), 100 mL. Mixture B. Epon 812, 100 mL; Methyl nadic anhydride (MNA) 89 mL. Although both mixtures can be stored at 4°C, the embedding mixture must be prepared on the day of use. To prevent the spoiling hydration of the held cold mixtures, leave them at room temperature before opening the bottles. We found that the harder mixture is the best for the crossbeam electron microscope: Thus, 10 mL of the final embedding mixture (FEM) by mixing thoroughly 3 mL of Mixture A, 7 mL of Mixture B and 0.2 mL of the accelerator DMP30.

Dehydration and gradual resin infiltration of tissue samples with the embedding mixture is shown in Table 1.

**TABLE 1:** FEM: Final embedding mixture. Mixture A: Epon 812, 62 mL + dodecenyl succinic anhydride (DDSA), 100 mL. Mixture B: Epon 812, 100 mL + methyl nadic anhydride (MNA), 89 mL.

Step #	Solution	Time	Temp
**1. Dehydration**	Acetone 25%	15 min each	Room Temp
	Acetone 50%		
	Acetone 75%		
	Acetone 95%		
**2. Resin preparation**	FEM		Room Temp
	Mixture A. 30%		
	Mixture B. 70%		
**3. Resin infiltration**	1.FEM+100% acetone, 1:2	1.0 Hour each	Room Temp (Rotating slowly)
	2.FEM+100% acetone, 1:1		
	3.FEM+100% acetone, 2:1		
**4. Resin Polymerization**	FEM + DMP30 2%	24 h	60 °C
	FEM polymerization 24 h		

## Stepwise trimming of embedded samples for light and electron microscopy

**FIGURE 1:**
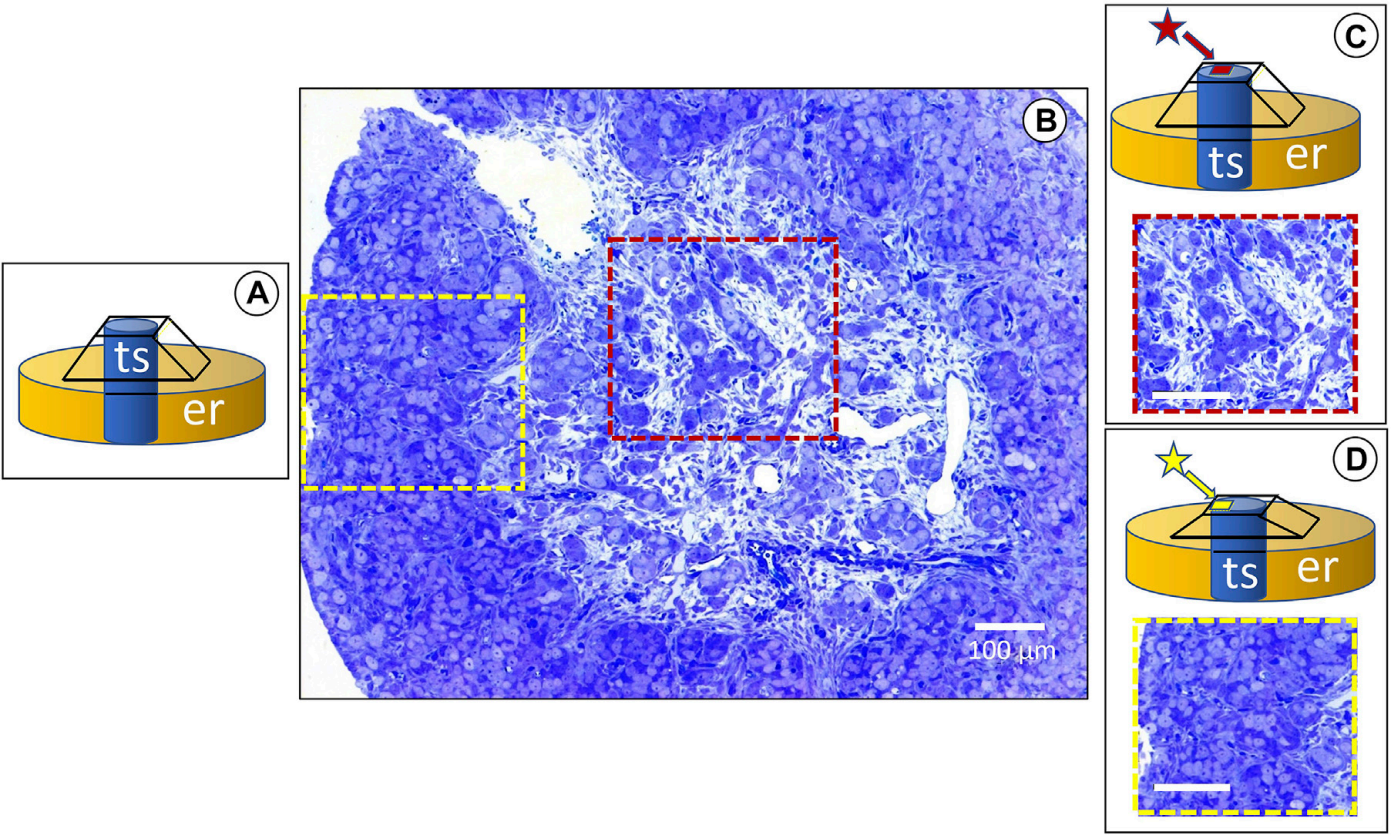
The red and yellow dashed squares show the medullary and cortical regions consecutively, analyzed by light and FIB-SEM. The insets **(A–D)** are schematic representations of the serial trimming procedure: **(A)** the tissue sample (ts) embedded in the resin (er) after a pyramid was formed from a razor blade on the top of the block. **(B)** Cross section of a developing rabbit ovary at 25 days (dpp) postpartum, stained with toluidine blue. The histological differences between the medullary and cortical regions are evident. The former has abundant loose stromal tissue around the ovigerous cords, whereas the latter shows a compact structure. **(C)** A square around 1.0 mm high (red star) is manually constructed on top of the truncated pyramid, in order to make serial semithin sections for 3D reconstruction of the medullary region. Under the light microscope. **(D)** Once an area of interest is found in the medulla, a similar procedure is performed on the ovarian cortex (yellow star), in order to analyze diverse regions of the ovary as relates to the FIB-SEM cycles of semithin serial sections and then undertake stepwise trimming at the ultramicrotome.

Routine 2D imaging of resin-embedded samples for TEM requires consecutive semithin (around 1 µm) and thin sections (about 700 nm) for LM and TEM, respectively. Ultramicrotome semithin sections can be obtained using glass or diamond knives and block surface areas as large as two square millimeters. Then, sections stained with toluidine blue allow the structural observation of tissues at the LM’s highest resolution limit. A pyramid is carved by hand on the surface of the block (Figure 1A). In complex tissues of developing organs, stromal and epithelial cells undergo a continuous spatiotemporal change. Thus, the ultrastructural analysis of the area of interest was usually made by carving a pyramid-like shape on the block surface at different locations (Figure 1B). A plate of around 0.5 mm can be engraved onto the site of interest, to save precious tissue around the truncated top of the pyramid (Figure 1C). By alternating semithin and thin sections throughout a developing organ, high-resolution 3D light microscope analysis of tissues can be undertaken at diverse regions of interest. The above-described workflow can be extended to analyze both LM and FIB-SEM tissue volume. Volume reconstructions can be made at the highest resolution of the LM using the block edges and morphological cues (as blood vessels) as a reference. Once the area of interest is located on a large semithin section stained with toluidine blue (Figure 1), semithin serial sections can be obtained from the carved plate on the block surface. Moreover, thin sections for the TEM can be taken before or after the serial semithin sections. Most developing organs show regions with stromal and epithelial tissues at different stages of structural differentiation. A differentiation gradient among the cortical and medullary regions is evident in the ovary.

Furthermore, the present workflow allows the analysis at the FIB-SEM using the same block. Thus, the volume of regions of interest at the resolution of the SEM can also be accomplished.

## Focused ion beam scanning electron microscop tomography

**FIGURE 2:**
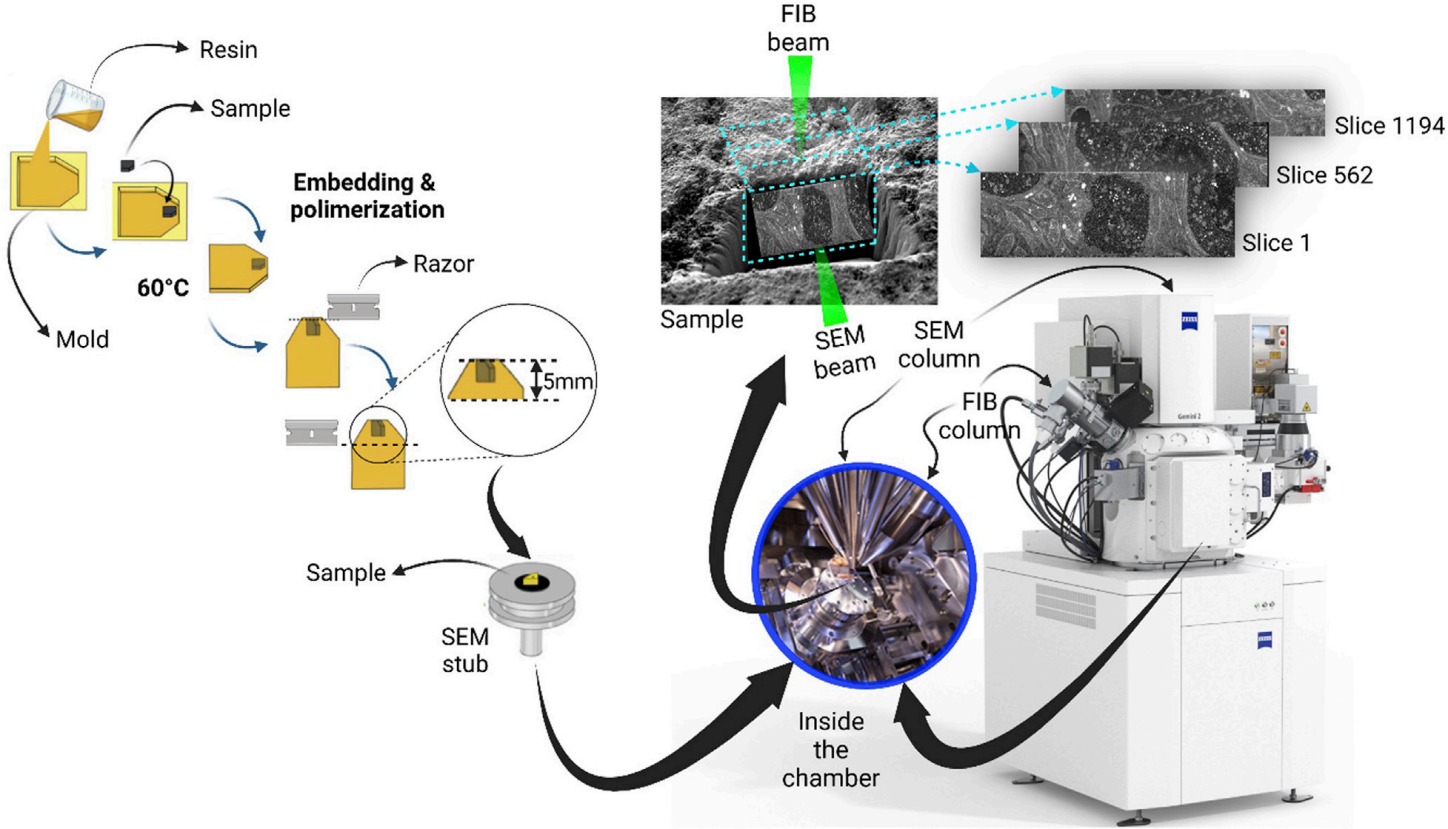
Workflow of sample preparation for focused ion beam milling and SEM imaging process, under a dual beam electron microscope. The ion source (FIB) and the electron source (SEM) are arranged at an angle (54°), allowing the ion beam (Ga+) to remove material from the surface of the specimen, so that the scanning electron beam can make it visible. As a result, a trench (light blue) is generated, thus making the specimen’s interior visible. As shown, the exposed surface is parallel to the plane of the ion beam and angled (54°) to the electron beam.

A general overview of the FIB-SEM method is shown in Figure 2. A FIB-SEM ZEISS Crossbeam 550 instrument (Zeiss Group, Oberkochen, Germany) equipped with a high-performance field emission beam with a gallium-ion beam-column. The ion beam accelerated at 30 kV was used for both raw and fine milling: an ion current of 15 nA was applied to make a coarse trench around the protective Pt layer. Once located an area of interest on the trimmed surface of the block, it was sputter coated with gold after being fixed on an aluminum stub (20 mm^2^) size with colloidal silver. A thin platinum film preventing a curtain effect was deposited on the top of the catalytic layer by injecting a platinum precursor from the gas injection system, the platinum was placed using a beam condition of 30 kV, 1.5 nA, and a Dwell time of 0.1 µs. The resulting movie’s approximate thickness of 1 μm covered an area of 10 × 10 μm. To avoid a misalignment issue caused by the beam shift, the sample drift, or both.

BSE images of the exposed planes were obtained at an accelerating voltage of 5 kV and a current of 0.12 nA. In contrast, a current of 1.5 nA was optimal for the sequential removal of thin slices of the tomography process. The total depth of the trench formed by this procedure was 20 µm.

The orthogonal coordinates were chosen so that backscattered electron images were in the X-Y plane while the sequential removal of slices went along the third axis. After tentative image processing, a pixel size of 20 nm × 10 nm was used for tomography, which conformed to a slice thickness of 20 nm. It is possible to work directly with volume images consisting of cubic voxels. Raw FIB-SEM of 2138 images, cropped and processed to show all images collected as a single volume image. This process was done using ORS Dragonfly Software.

## Results

**FIGURE 3:**
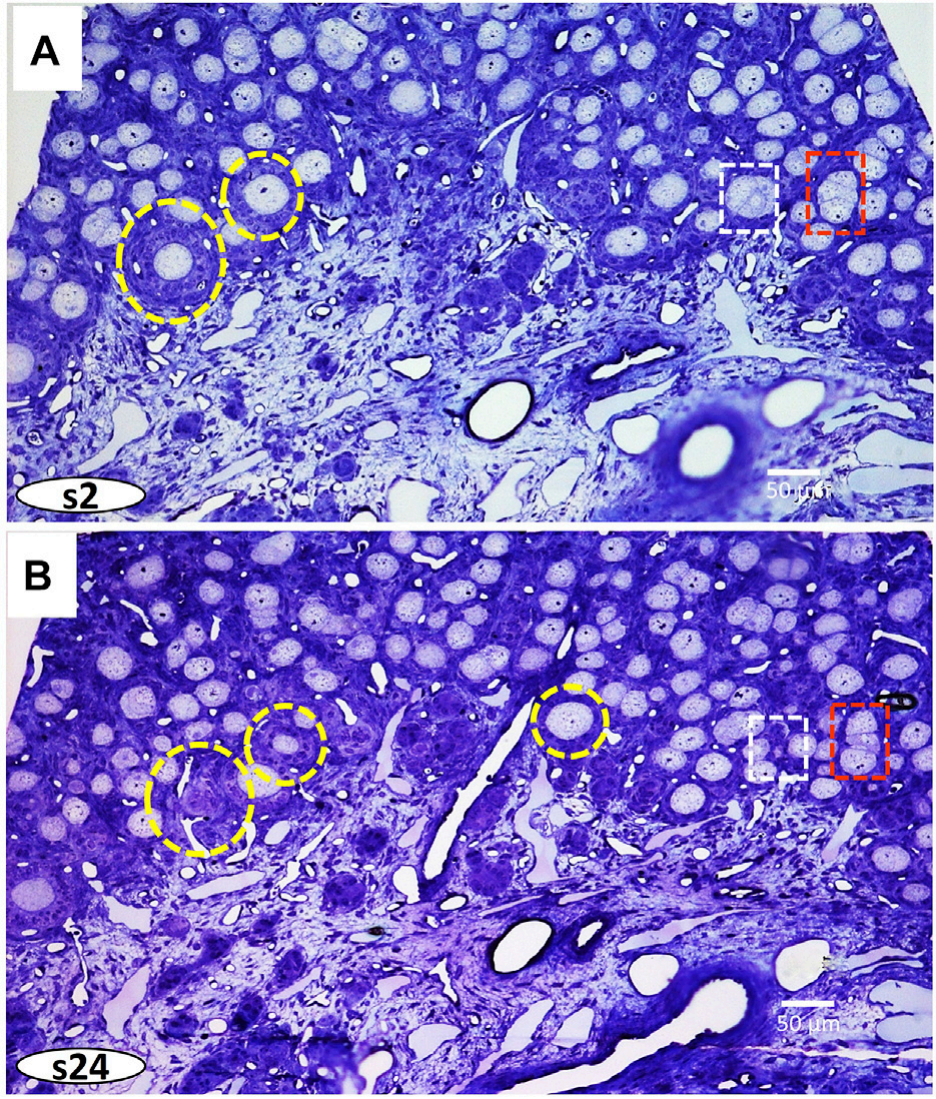
Two images of semithin sections (1.0 µm) at different levels: **(A)** section two (s2), and **(B)** section twenty-four (s24), were taken from Supplementary Movie S1 (Supplementary Material). Dashed circles and squares correspond to Follicles and cysts, respectively. Follicles represent individual oocytes enveloped within follicular cells. Ovarian cysts are formed by clusters of oocytes at different stages of fusing within the ovigerous cords.

Serial semithin sections (1.0 µm) allow 3D reconstructions of regions of interest at the histological level through the block. Thus, diverse areas can be selected at the XY and Z planes. The volume reconstruction of 56 sections of the region is shown in the S1 movie, directed in the supplementary material. Figure 3 shows the images of two areas taken at different levels of the Z plane. On these images, diverse developmental steps of folliculogenesis can be marked at plane XY at different levels and then reconstructed in-plane Z through the embedded sample.

**FIGURE 4:**
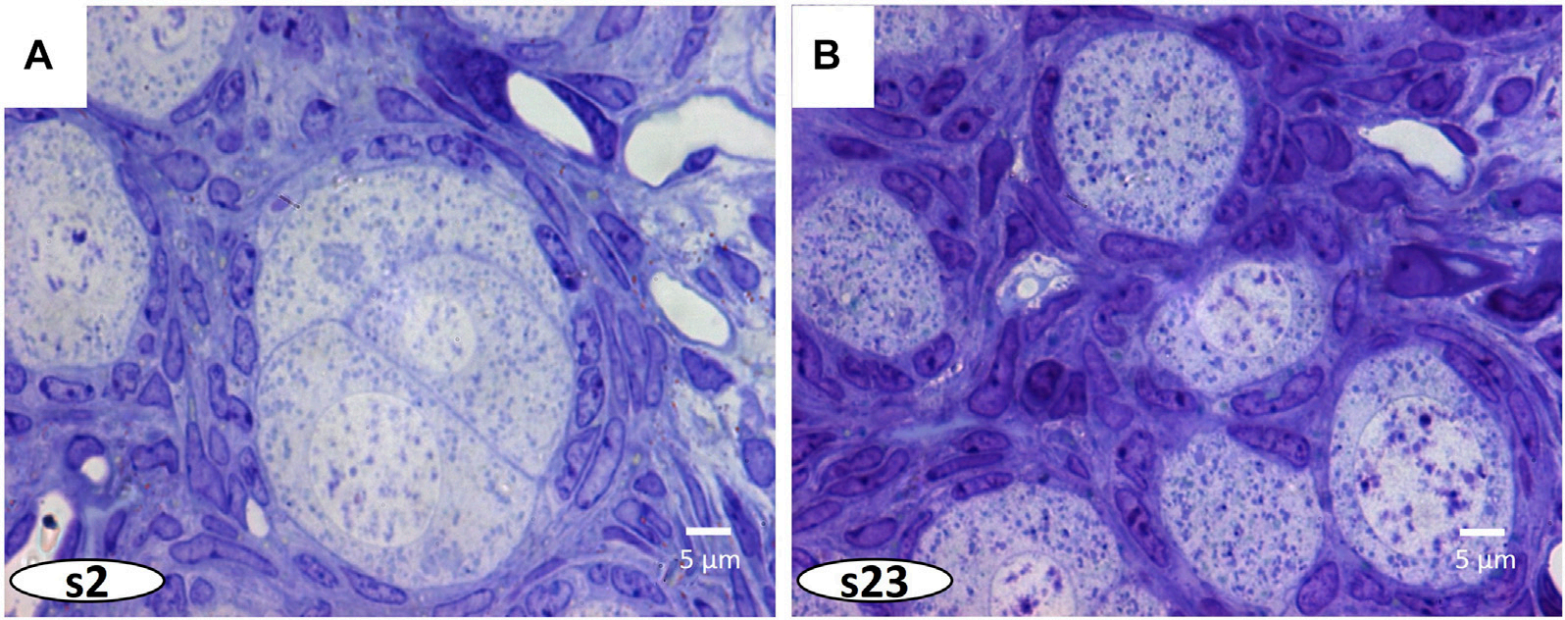
In Figure 3, a greater magnification of the ovarian cyst is indicated by a dashed white square. **(A, B)** The 3D reconstruction of this cyst shows close association between oocytes and surrounding pre-follicular cells.

Cytoplasmic organelles and meiotic chromosomes are seen in the germ cells, and diverse somatic cells and blood vessels can be identified. Moreover, the marked regions of interest can be analyzed at the highest resolution of the light microscope and 3D reconstructed. (Figure 4 and Supplementary Movie S2).

**FIGURE 5:**
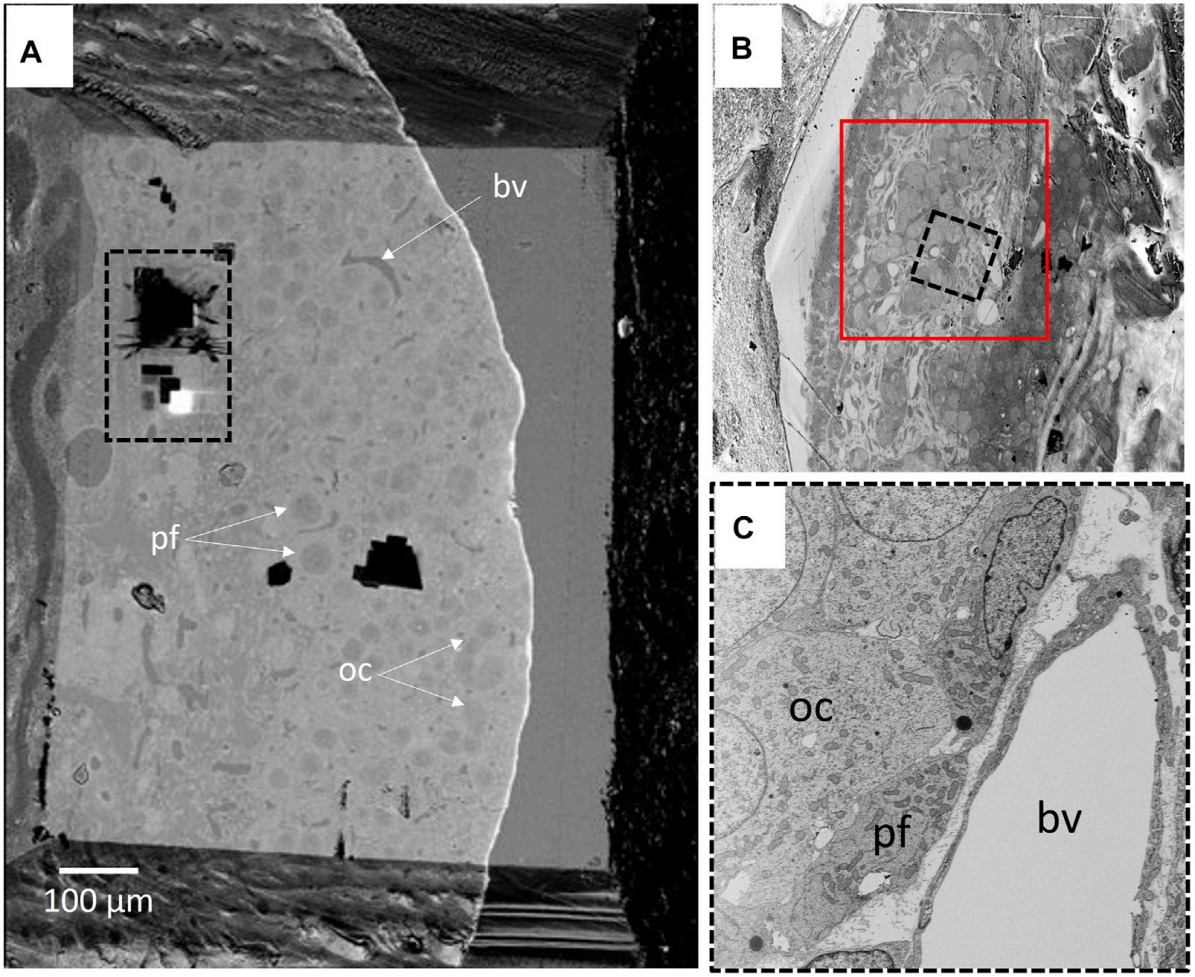
**(A)** BSD4 block face overview, showing part of the ovarian cortex and part of the medullary region. Ovarian cysts and primary follicles are visible. The black dashed rectangle delineates the 3D run position shown in Supplementary Movie S2 (Supplementary Material). **(B)** The upper right inset shows the cortical region of the ovarian cortex (red square) on which the area of interest. **(C)** An image of the 3D run region is shown in the lower right inset. Oocyte (oc) and pre-follicular cells (pf) of an ovarian cyst were easily located. The blood vessel (bv) was taken as a point of reference.

**FIGURE 6:**
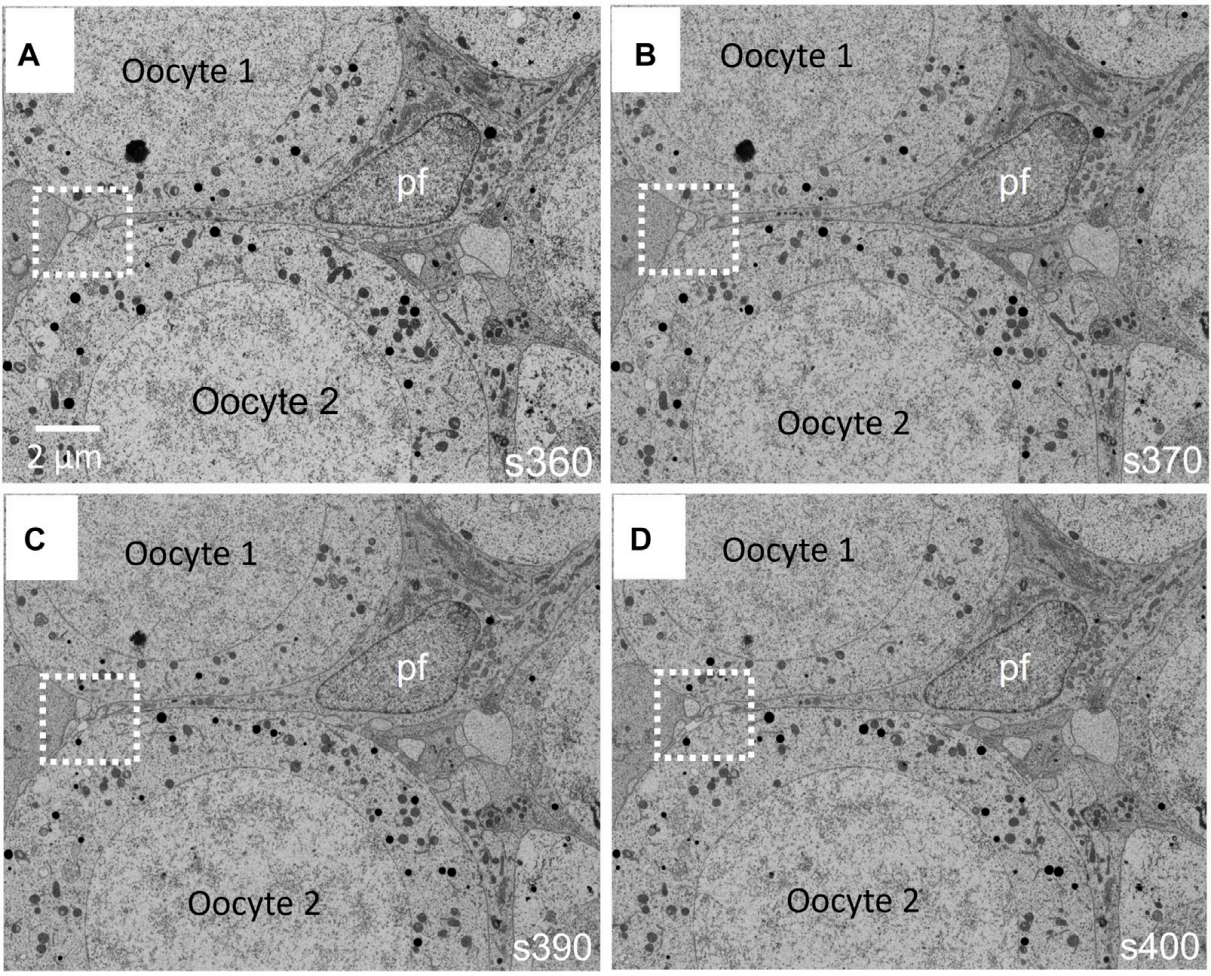
**(A–D)** Four images taken between runs 360 and 400 (s360–s400) with the FIB-SEM from a total of 1300 runs are shown in Supplementary Movie S3 (Supplementary Material). Prior to oocyte (oc) fusion within the cyst, these are separated by pre-follicular cells (pf). Interestingly, the oocytes undergo filopodia-like processes, which go through the follicular cells, making possible direct contact between oocytes (white dashed square). These filopodia-like processes have not been described before.

**FIGURE 7:**
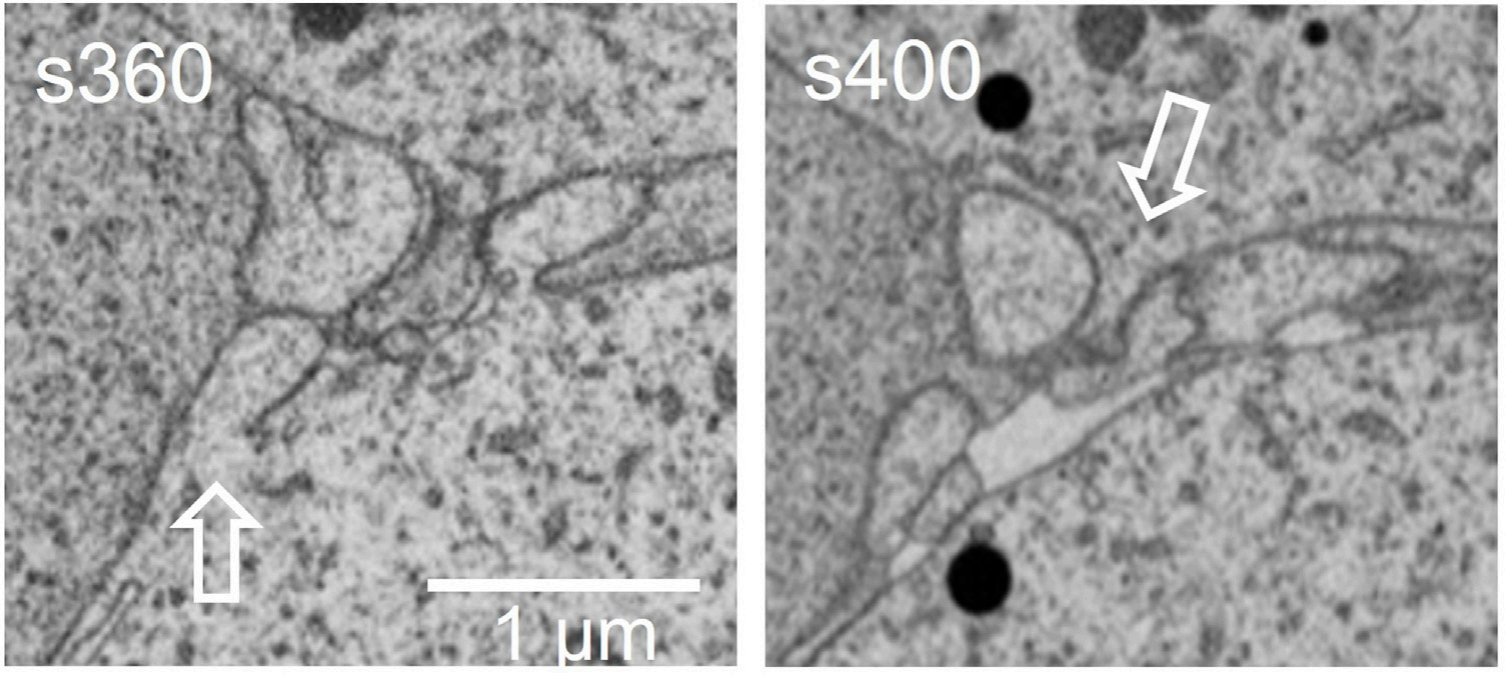
This is an amplified magnification of the region shown in the dashed square of Figure 6. The pre-follicular cells (pf) were marked in Figure 6. The dashed box, visible in the movie shows the filopodia-like extensions, where oocytes make contact.

At the FIB-SEM, the block face overview shows cues identified at the light microscope (Figure 5). The focused ion beam milling and scanning electron microscope imagining at an angle 54° generate trenches which enable the 3D analysis of tissues in different locations of the area of interest. Figure 6 illustrates backscattered electron images of four serial slices, taken between slices s360 and s400 from a total of 1,300 slices of a thickness of 20 nm (see Supplemntary Movies S3). The ultrastructure of cell organelles in both germ and somatic cells and the tissue arrangement enables the 3D study of cell-cell interactions at different developmental stages. Here we found thin cytoplasmic processes (filopodia-like) interconnecting the oocytes through the somatic cells within the ovarian cysts (white dashed square in Figure 7 and Supplementary Movie S3). These cytoplasmic oocyte processes have not been seen before. They suggest that a complex oocyte-oocyte interaction occurs before their fusion in mammalian ovaries’ cysts (Lei and Spradling, 2016).

Primordial follicular reserve (PFR) in mammalian ovaries is essential for female fertility. The establishment of the PFR occurs as a stepwise process in developing ovaries. Germ cell differentiation is closely related to pregranulosa somatic cells within the ovigerous cords in the ovarian cortex. Initially, mitotic oogonia form clusters of interconnected germ cells known as cysts, which separate to form the primordial follicles. The differentiation process takes place as a regional gradient from the outer to the inner ovarian cortex during periods of days, weeks, or months, depending on the species. In the rabbit, PFR is established after about 35 days (Díaz-Hernández et al., 2019) (Table 2). Individualized follicles start to form from a surviving oocyte in each cyst, which grows thanks to the transfer of cell organelles from sister oocytes that die from apoptosis (Lei and Spradling, 2016). The cellular and molecular mechanisms underlying these complex processes in mammals remain poorly understood.

**TABLE 2:** Time scale of postnatal rabbit ovaries at which cysts and follicles are formed. The breakdown of rabbit cysts takes place in a stepwise fashion over 35 days. DPP: days postpartum.

DPP	Outer cortex	Inner cortex	Histology
1	Oogonia	Oogonia and Cysts	Ovigerous cords
7	Oogonia and cysts	Meiotic Cysts	Ovigerous cords
14	Mitotic and meiotic cysts	Meiotic Cysts	Ovigerous cords start
		First Follicles	breaking down
28	Mitotic and meiotic cysts	Meiotic Cysts	Most ovigerous cords
		Follicles	Break down
35	Meiotic cysts	Follicles	Few ovigerous cords

## Discussion

This paper presents a practical and reliable workflow to volume imaging of large tissue samples, using light microscopy and FIB-SEM. Our protocol makes possible structural and ultrastructural analysis of wide areas using morphological cues. Thus, volume imaging of cells and tissues, located at different locations in developing organs can be analyzed at consecutive stages of development. Combined with well-established tissue patterns of confocal immunofluorescence, our workflow can reveal the underlying ultrastructure. Furthermore, a similar approach may be useful to study normal and pathological samples of adult tissues.

The current protocol manifests the intrinsic limitations that the TEM protocols have for optimal preservation of tissue samples: 1. Fixative solution must reach living tissue as quickly as possible, for which systemic perfusion is better than immersion. 2. The size of the samples should not exceed 2 mm in thickness both to fix the samples by immersion and to allow complete penetration of heavy metals. The Epon 812 used in this protocol is harder than that usually used for TEM. Although glass knives can be used for semithin sections, if the highest resolution of TEM is required, it will be necessary to use a costly diamond knife. However, the heavy deposit of metals in the sample increases the risk of damaging the diamond knife.

Recent technological advances in light and electron microscopy and imaging the molecular structure of cell organelles led to correlative light and electron microscopy (CLEM). Most methods mainly use *in vitro* systems: cells, tissues, embryoid bodies; or tiny model organisms: Drosophila, *C. elegans*, zebra fish, etc (Caplan et al., 2011; de Boer et al., 2015; Bykov et al., 2016; Porrati et al., 2019). There is increasing evidence indicating that several regulatory gene networks according to Eric H. Davidson’s concept involved in cell differentiation and morphogenesis are generally conserved among different taxa (Davidson, 2006). However, the spatiotemporal mechanisms involved in organ morphogenesis evolved according to the increasing complexity of the species. Thus, understanding the molecular mechanisms underlying the process of organogenesis, requires the integration of spatiotemporal data from at least three layers of complexity: molecular, cellular and tissular.

Classical descriptive and experimental embryology relayed in sampling embryos at consecutive developmental stages, adding the time dimension to the structural three dimensions. We consider that the ultrastructure of cells and tissues established by the TEM remains valid as important reference to the emerging technological advances aimed to elucidate the molecular architecture of the cell organelles. Even though the actual workflow is limited to descriptive data using only morphological cues, it reveals volume reconstructions at various levels of complexity: cell organelles, cells, tissues, and organs. Once established the structural volume pattern of tissues in a given organ with the light microscope, the proposed workflow is a simple method that can be combined with the high resolution of the FIB-SEM.

## Data Availability

The original contributions presented in the study are included in the article/Supplementary Material, further inquiries can be directed to the corresponding author.
