# Supplementary material for: Light and focused ion beam microscopy workflow for resin-embedded tissues
Source: Front Cell Dev Biol. 2023 Jan 25;11:1076736. doi: 10.3389/fcell.2023.1076736 (PMC9905623; doi:10.3389/fcell.2023.1076736)
Supplement: Supplementary file 2 [file Table1.DOCX]

Chemical products Catalog number Company

Acetone 10000 EMS (Electron Microscopy Science)

Acetonitrile 10020 EMS

EMbed 812 Kit 14120 EMS

Ethyl Alcohol 15058 EMS

Glutaraldehyde 16110 EMS

Lead acetate 2271-05 J.T. Baker

Malachite green 18100 EMS

Osmium Tetroxide 201030 Sigma-Aldrich

Paraformaldehyde P6148 Sigma-Aldrich

Sodium Cacodylate Trihydrate 12310 EMS

Uranyl acetate 22400 EMS
